# Synchrotron infrared imaging of advanced glycation endproducts (AGEs) in cardiac tissue from mice fed high glycemic diets

**DOI:** 10.3233/BSI-130057

**Published:** 2013

**Authors:** Giovanni Birarda, Elizabeth A. Holman, Shang Fu, Karen Weikel, Ping Hu, Francis G. Blankenberg, Hoi-Ying Holman, Allen Taylor

**Affiliations:** a Berkeley Synchrotron Infrared Structural Biology Program, Lawrence Berkeley National Laboratory, University of California, Berkeley, CA, USA; b Department of Radiology and Pediatrics/Molecular Imaging Program at Stanford, Stanford, CA, USA; c Laboratory for Nutrition and Vision Research, Jean Mayer USDA HNRCA at Tufts University, Tufts University, Boston, MA, USA; d Boston Medical Center, Boston University School of Medicine, Boston, MA, USA

**Keywords:** AGE, CVD, diabetes, fluorescence, glycemia, glycation, infrared, N•-(carboxymethyl)lysine, PAGE, pentosidine, RAGE, sugar

## Abstract

Recent research findings correlate an increased risk for dieases such as diabetes, macular degeneration and cardiovascular disease (CVD) with diets that rapidly raise the blood sugar levels; these diets are known as high glycemic index (GI) diets which include white breads, sodas and sweet deserts. Lower glycemia diets are usually rich in fruits, non-starchy vegetables and whole grain products. The goal of our study was to compare and contrast the effects of a low vs. high glycemic diet using the biochemical composition and microstructure of the heart. The improved spatial resolution and signal-to-noise for SR-FTIR obtained through the coupling of the bright synchrotron infrared photon source to an infrared spectral microscope enabled the molecular-level observation of diet-related changes within unfixed fresh frozen histologic sections of mouse cardiac tissue. High and low glycemic index (GI) diets were started at the age of five-months and continued for one year, with the diets only differing in their starch distribution (high GI diet = 100% amylopectin versus low GI diet = 30% amylopectin/70% amylose). Serial cryosections of cardiac tissue for SR-FTIR imaging alternated with adjacent hematoxylin and eosin (H&E) stained sections allowed not only fine-scale chemical analyses of glycogen and glycolipid accumulation along a vein as well as protein glycation hotspots co-localizing with collagen cold spots but also the tracking of morphological differences occurring in tandem with these chemical changes. As a result of the bright synchrotron infrared photon source coupling, we were able to provide significant molecular evidence for a positive correlation between protein glycation and collagen degradation in our mouse model. Our results bring a new insight not only to the effects of long-term GI dietary practices of the public but also to the molecular and chemical foundation behind the cardiovascular disease pathogenesis commonly seen in diabetic patients.

## 1. Introduction

Spectral imaging using infrared radiation emitted from a thermal emission light source has emerged as a routine chemical imaging tool permitting the identification of the fingerprint-like mid-infrared spectra of macromolecules in histologic tissue samples both from animal models and humans [14,18,70]. Spectral features such as peak frequency, shape, and intensity are directly related to the spatial distribution of the intrinsic chemical compounds. For example, FTIR spectral microscopy is routinely used to assess the relative abundance of phosphate, carbonate and collagen in mineralized tissues, providing a valuable measure of mineral quantity and quality [7,9]. FTIR spectral microscopy has been applied to study diet-induced atherosclerotic lesions in the descending thoracic segment of rabbit aorta [55] as well as drug- and disease-associated effects on the cellular composition such as the lipid- and carbohydrate-to-protein ratio in tissues [39,44–46]. In these studies, the spectral features of the compounds of interest were readily resolved, and the compound distribution was evaluated by means of univariate analysis. In situations where the spectral features of targeted compounds overlap, either supervised or unsupervised multivariate data analysis was applied to extract semi-quantitative information on tissue components and their relative abundance [5,57,73].

Evaluation of large volumes of data reveals that improvements in spatial resolution and the intensity of the signal arriving at the detector could significantly improve the FTIR spectromicroscopy performance. Here spatial resolution determines the measurement area within the biological sample and therefore the length scale of the heterogeneity that can be studied. One limiting parameter is the intensity of the signals. It has been shown that both spatial resolution and signal-to-noise can be dramatically improved by replacing the thermal emission source in the conventional FTIR spectromicroscope with a bright synchrotron infrared source [17,34]. In 1998, synchrotron FTIR was first applied to map the distribution of macromolecules in a single human cell [36], and shortly afterwards to characterize human lung epithelial cells during the cell cycle and the early stage of apoptosis [32]. This was followed by a growing trend of diagnosis of tissues for biomedical applications which includes identifying the structures of misfolded protein aggregates in the brain tissue of Alzheimer’s disease patients [43] detecting the evolution of Huntington’s disease [6], characterizing the neuropathology of multiple sclerosis [30], infectious prions in scrapie-infected tissues [15,40] analyzing bone and cartilage disease [22,61], identifying the effect of high-fat diet on the severity of atherosclerosis, and to evaluate the progression of atheromatous plaques in mice [33]. More recently synchrotron infrared spectromicroscopy has been applied to the detection of metabolites in Purkinje neurons [25] as well as to track the changing biochemistry and protein phosphorylation of nerve cells as they differentiated [10].

In this study, synchrotron infrared spectromicroscopy was used to investigate the biochemical composition of cardiovascular tissues from older adult mice fed with a high glycemic index (GI) diet for one year. The glycemic index compares the two-hour area under the blood glucose curve due to consuming a food containing 50 grams of digestible carbohydrate to the two-hour area under the blood glucose curve due to consuming 50 grams of a standard carbohydrate (i.e. glucose, white bread). Low GI food has a GI value of 55 or less and high GI is 70 and above. Glucose has a GI of 100 [37]. Foods with a high GI induce a larger increase in blood glucose levels than foods with a low GI. Under normal physiological conditions, glucose reacts with proteins, lipids and nucleic acids through non-enzymatic glycation and oxidation to form a heterogeneous group of glycated molecules called advanced glycation endproducts (AGEs), with highly reactive dicarbonyl glyoxal compounds as intermediate products [8,12,29].

Glycated protein, lipid end-products and AGEs cross-link intracellular and extracellular matrix proteins, altering tissue function and biochemical and mechanical properties [29,68,71] as well as the controversial induction of collagenolysis [67]. AGEs also interact with a specific receptor present on all cells, known as RAGE (receptor for AGE). RAGEs are relevant to the development and progression of cardiovascular disease associated with the pathologic activation of a variety of cells including monocytes derived macrophages, endothelial cells, and smooth muscle cells. The interactions of AGEs with RAGE result in the induction of oxidative stress and pro-inflammatory responses, increase oxidative stress, and the activation of protein kinase C that alters the growth factor expression [3]. Increased levels of AGEs have been observed in serum of patients with chronic hyperglycemia due to diabetes mellitus (DM) (see references in [13,71,72]). The enhanced formation and accumulation of AGEs are believed to have a key role in the pathogenesis of cardiovascular disease, the leading cause of early death among people with DM. Approximately 65% of people with diabetes eventually die from diabetic heart disease, and diabetic adults are also two to four times more likely to have heart disease or suffer a stroke than the age adjusted normal population in the United States [54].

Similar to patients with chronic hyperglycemia due to DM, epidemiologic data indicate that people who consume high GI diets have a significantly increased risk for cardiovascular disease [49,51]. However, our understanding of the connection between a high GI diet and the accumulation of advanced protein and lipid modification products is far from complete; their roles in the transformation of collagen and the disruption of the stability of the cardiovascular tissues are unclear and controversial. In vitro methods of investigation used to date often rely on AGE-specific immunochemical tests (i.e. AGE-ELISA), but such assays cannot directly illustrate the spatial distributions of AGEs, collagen and lipids within a single tissue section. Immunohistochemical staining can also be used to identify the spatial distribution of AGEs [11,12,62,72], yet they lack the spatial information of other supporting biochemical processes, biomolecules and microstructures.

A number of investigators have demonstrated that tissue structure and function can be progressively modified by AGEs through the fluorescent pentosidine cross-links between the arginine and lysine residues in collagen as well as the nonfluorescent N*-(carboxymethyl)lysine that initiates an AGE receptor mediated effect [20,21,23,27–29,65,66]. In the study reported here, we have used synchrotron infrared spectromicroscopy, together with fluorescence microscopy to examine *in situ* the connection between AGEs, long-lived proteins such as collagen, and lipid peroxidation. In parallel, we also performed hematoxylin–eosin (H&E) analysis of adjacent tissues in serially sectioned heart tissues to correlate the chemical information with the histopathologic information.

## 2. Materials and methods

### 2.1. Ethical considerations

This study was carried out and approved under the Jean Mayer United States Department of Agriculture Human Nutrition Research Center on Aging at Tufts University Institutional Animal Care and Use Committee protocols, in accordance with the Animal Welfare Act provisions and the ARVO Statement for the Use of Animals in Ophthalmic and Vision Research and with all other animal welfare guidelines, such as the National Institutes of Health Guide for the Care and Use of Laboratory Animals.

### 2.2. Animals

Cardiac tissues from a C57BL/6 nontransgenic mouse model, fed a high or low GI diet were used for our current work. Five-month-old male C57BL/6 mice (approximates middle aged) were obtained from Charles River Laboratories (Wilmington, MA). The mice were divided into 2 groups. Mice in group 1 were fed a high glycemic index (GI) diet (100% amylopectin starch) and mice in group 2 were fed low GI diet (30% amylopectin/70% amylose starch) for 12 months. The mice were pair-fed to ensure equal consumption between diet groups. Diets were isocaloric and of identical macronutrient distribution (65% carbohydrate, 21% protein, 14% fat). The only difference between the high and low GI diets was the distribution of starch (100% amylopectin in the high GI diet, and 30% amylopectin/70% amylose in the low GI diet). All of the diets used in this study were formulated by Bio-Serv (Frenchtown, NJ). National Starch (Bridgewater, NJ, now Ingredion) generously donated Amioca starch (100% amylopectin) for incorporation into the high GI diet, and Hylon VII starch (30% amylopectin/70% amylose) for incorporation into the low GI diet. At 17 months of age, the mice were fasted 6 hours prior to being euthanized with carbon dioxide. Tissues were then harvested and frozen immediately in liquid nitrogen and stored long term at −80°C.

### 2.3. Tissue preparation

Each frozen sample was serially cryosectioned at a thickness of 5-micrometers free of freezing media on the cut surface using liquid nitrogen-isopentane cryogens at the Vogel Lab at Stanford University. The odd numbered sections were mounted on a silicon slide for infrared spectromicroscopic analysis. Even numbered sections were mounted on a glass slide and stained with hematoxylin and eosin (H&E) for light microscopic analysis. H&E has traditionally been used to highlight the morphology and microstructure (i.e. normal histology and histopathology) of the tissue sections. It was not possible to correlate SR-FTIR imaging and H&E staining on the same slide because the H&E preparation procedure alters the infrared absorption characteristics of a tissue section.

### 2.4. H&E staining of thin sections for light microscopy

The even-numbered thin tissue sections (from 2.2) were taken straight from the cryotome and briefly set to dry on warm glass slides before being placed in hematoxylin for 5 min, rinsed, placed in eosin for a minute, dehydrated with graded strengths of alcohol, cleared in xylene, and finally cover-slipped using a permanent mounting medium. The stained sections were examined using a standard light microscope.

### 2.5. Synchrotron infrared spectral microscopy measurements

*In situ* measurements of the non-stained odd-numbered tissue sections were performed in biological triplicate using a Nicolet Continuum infrared microscope with a mercury cadmium telluride (MCT) single element detector (Thermo Fisher Scientific Inc.). The detector was connected to a Nicolet 6700 FTIR spectrometer coupled with a synchrotron light source at the infrared beamline 5.4 (Advanced Light Source, Lawrence Berkeley National Laboratory; see http://infrared.als.lbl.gov/). The unfixed 5 μm thick cryosections of the tissues from mice fed with different GI diets were placed under a 32X objective with a numerical aperture (NA) of 0.65. The tissues were sampled by dividing the whole area into 10 μm pixels and then raster scanned, collecting full SR-FTIR spectra at each position. All spectra were recorded over the mid-infrared region in transmission mode, and each spectrum represents an average of 8 scans with a spectral resolution of 4 cm^−1^ (Thermo Fisher Scientific Inc.). The resulting data cubes, which consisted of position-associated FTIR spectra, were saved in ENVI format and imported in “R” environment using hyperSpec (http://hyperspec.r-forge.r-project.org).

### 2.6. Synchrotron infrared spectral data analysis

The hyperSpec package in “R” environment was used for spectral processing such as baseline correction and detection of void areas in the tissue, for calculating the derivatives, and for peak intensity analysis (Figs 2 and 3). The void areas in the samples were identified by cluster analysis and masked during the SR-FTIR analysis. The peak intensity analysis, which integrates infrared absorbance of an individual peak of interest, relates the absorbance intensity at a given frequency *ν* to the relative concentration of a particular chemical component and the thickness of the sample through the Beer–Lambert Law. In this study, the thickness of sample section was near constant. The integrated area of the amide I and amide II (1700–1480 cm^−1^) absorption bands that arise mostly from the combined vibrational modes of the C=O and O=C–N bonds of proteins (see Table 1) were mapped across the tissue section, creating an intensity image of proteins. Similarly, the integrated areas of the absorption band centered at *~*1025 cm^−1^, arising mainly from the vibrational modes of –CH_2_OH groups, and the absorption band centered at *~*1050 cm^−1^, arising mostly with the C–O stretching coupled with the C–O bending of the C–OH groups in carbohydrates, were mapped across the tissue section to create an intensity image of carbohydrates. The value of the lipids (2800–3000 cm^−1^), is mostly due to the symmetric and asymmetric stretching modes of methylene (CH_2_) and methyl (CH_3_) groups. In addition, the relative abundance expressed in terms of the ratio of carbohydrates to proteins signals were used to highlight sugar rich areas, where glycated proteins and precursors of AGEs (PAGEs) were likely present. Collagen I and III, the most abundant collagen subtypes in the heart, was evaluated using the *ν*(C–O) band centered at *~*1204 cm^−1^ [48]. The linear combination of the *ν*(C= O)_ester_ bands at 1730 and 1745 cm ^−1^ was used for lipid esters content [19].

### 2.7. Fluorescence microscopy analysis

To detect tissue structure that had been progressively modified by AGEs through the fluorescent pentosidine cross-links between the arginine and lysine residues in collagen, the tissue samples were also imaged by Fluorescence Illuminator equipped with the Nicolet Continuum microscope. This illuminator features a high-pressure mercury burner with a 12 V, 100-watt halogen bulb, and Interchangeable wide-band fluorescence cubes, which provide different excitation wavelengths: WU (330–385 nm excitation/*>*420 nm emission), BV (400–440 nm excitation/*>*475 nm emission), EN (450–490 nm excitation/500–550 nm emission), WB (450–480 nm excitation/*>*515 nm emission), WG (510–550 nm excitation/*>*590 nm emission).

## 3. Results

**Fig. 1:**
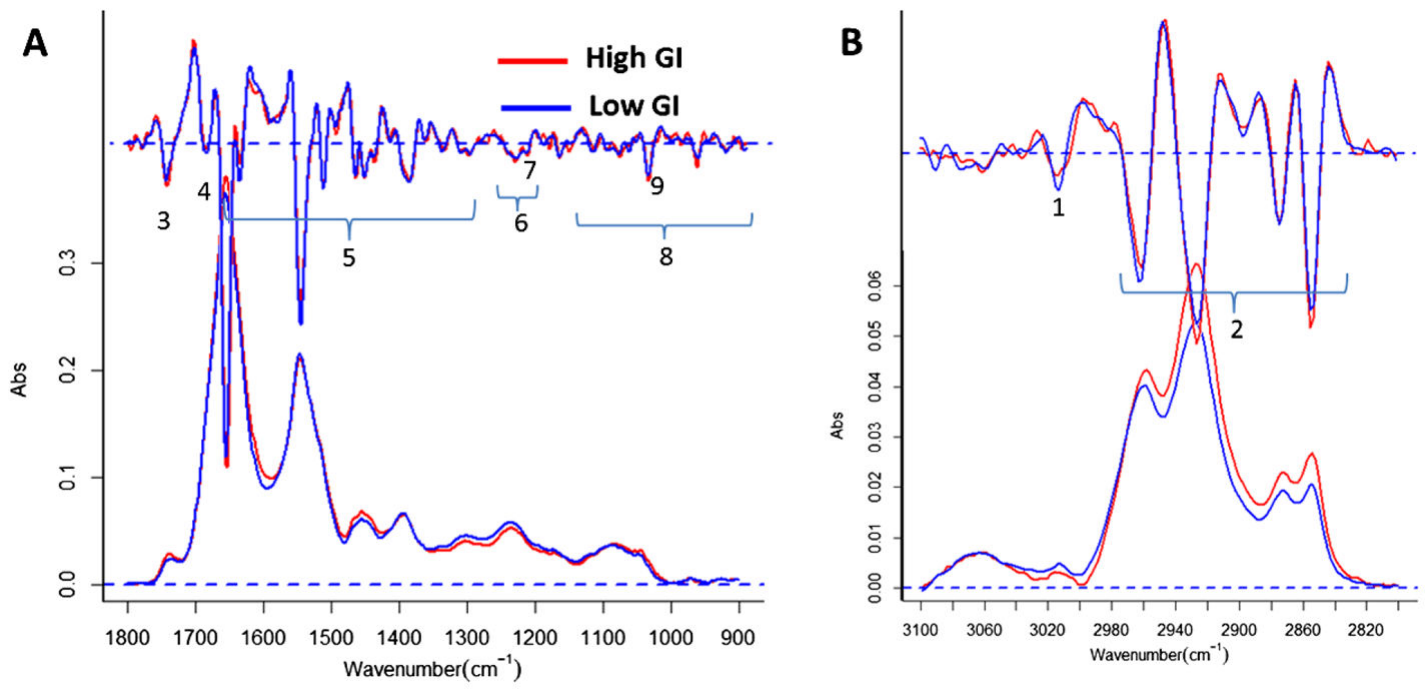
Average SR-FTIR absorption spectra of ventricular myocardium sections from mice fed high GI or low GI diets. Absorption and staked second derivative (A) in the 1800–1480 cm^−1^ protein amides and lipid ester region, and the 1480–900 cm^−1^ biomolecule fingerprint region, and (B) in the 2800–3100 cm^−1^ lipid region. See Table 1 and text for band assignments of major absorption frequencies (in wavenumbers). (Colors are visible in the online version of the article; http://dx.doi.org/10.3233/BSI-130057.)

A total of over 15,000 SR-FTIR spectra of ventricular myocardial tissue sections from both mouse groups were acquired over the mid-infrared (4000–650 cm^−1^) region. Their average absorption and second derivative spectra were analyzed in the 2800–3050 cm^−1^ lipid region, the 1800–1480 cm^−1^ protein amides and lipid ester region, and the 1480–900 cm^−1^ biomolecule fingerprint region (Fig. 1). The key spectral bands are labeled and their assignments are given in Table 1. In the 1800–900 cm^−1^ region, the High GI ventricular myocardium has a mean and a secondary spectrum similar to those of the low GI ventricular myocardium, except for a slight increase in the absorption intensity of the lipid ester, the protein amide I and the carbohydrate bands. However, the absorption intensities of the bands in the 2800–3050 cm^−1^ region that originate from the stretching vibrations of the fatty acids of all cellular lipids are distinctly stronger for mice in high GI diet, indicating that the relative lipid content in the tissues has increased compared to that for mice in low GI diet.

### 3.1. Global cardiac tissue composition: High versus low GI diet

The relative abundance of proteins, lipids, carbohydrates, collagens and cholesterols were estimated by using the Beer–Lambert Law, and expressed in terms of integrated infrared absorbance (in absorbance units, a.u.) of the main functional groups of the key macromolecules as given in Table 1. According to Beer–Lambert Law, the absorbance of spectral bands is proportional to the corresponding concentrations of chemical components. However, the actual optical path length of the sample may vary slightly as a result of variation of refractive index, therefore the values should be considered as an approximation. The estimated changes in their absorbance intensities are given in Table 2, which includes the integrated absorbance intensities of the amide I and II peptide bands (1700–1480 cm^−1^), the *ν* (CH) lipid bands (2800–3000 cm^−1^), the carbohydrate bands (1100–1000 cm^−1^), the *ν* (C–O) collagen band centered at 1204 cm^−1^ and the *ν*(C=O)_ester_ lipid ester bands. As shown in Table 2, the observed changes in proteins, lipids, carbohydrates, and lipid esters are statistically significant with diet. There is an overall 15% increase in the fatty acids/lipids content in the high GI diet groups, from 2.23 a.u. to 3.03 a.u. Similar increases in the lipid content was reported in the myocardium and vessels from patients with DM [60,69]. We observed little change in the collagen content with diet. The significance of the difference in fatty acids/lipids content with diet was confirmed by the ANOVA test on the complete dataset, with a *p*-value less than 3*×*10^−8^ (Tukey Multiple Comparison of Means). This suggested that a high GI diet could give rise to a higher lipid content within the cardiac tissues.

### 3.2. SR-FTIR imaging of biochemical changes

**Fig. 2:**
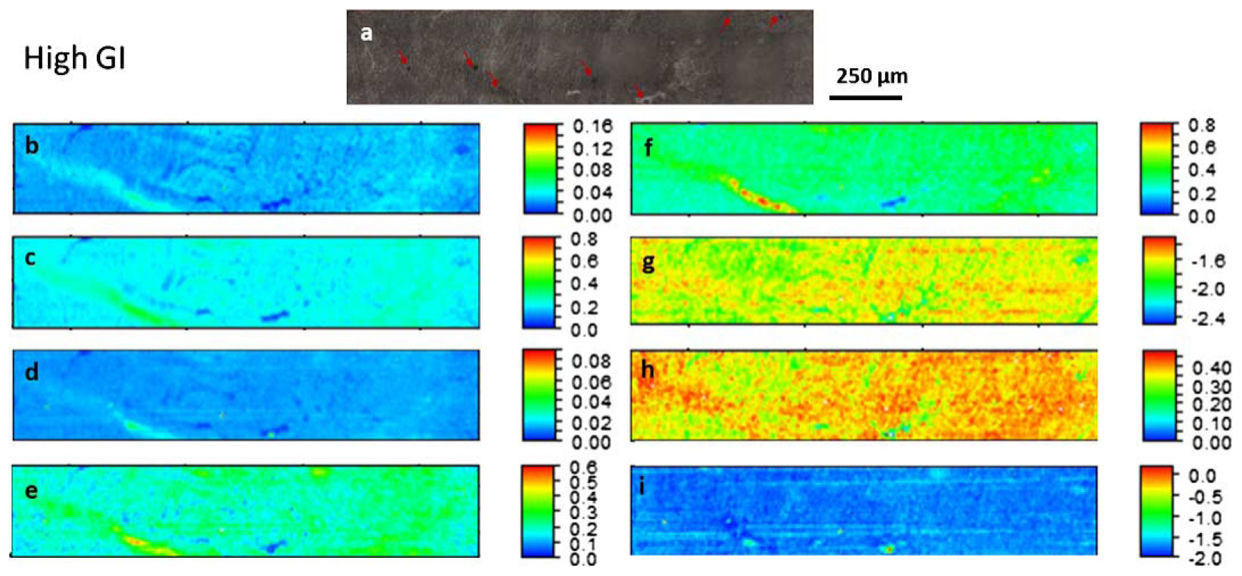
Bright field image and SR-FTIR heatmaps of a typical thin section of heart tissue from high GI-fed. (a) Optical image of cardiac tissue sections before the SR-FITR measurements. The SR-FTIR heatmaps are the spatial distribution of the integrated absorbance of (b) *ν*(C–H)_lipids_ and (c) amide I and II bands in the range of 2800–3000 cm ^−1^ and 1700–1480 cm^−1^ respectively. (d) Integrated absorbance of sugar moieties of carbohydrates, (e) *ν*(C–O)_collagens_ and (f) *ν*(C=O)_ester_ in the range of 1100–900 cm^−1^, at *~*1204 cm^−1^, and in the range of 1760–1700 cm^−1^ respectively. (g) The spatial distribution of the ratio of the integrated absorbance of *ν*(C–H)_lipids_ to protein amide I and II bands, of *ν*(CH_2_) to *ν*(CH_3_) of the fatty acids (h), and of the sugar moieties of carbohydrates to protein amide I and II bands (i). The elevated ratio of the carbohydrate bands to protein amide II reveal the presence of advanced glycation endproducts (AGEs). Note: All SR-FTIR heatmaps are pseudo-color images with the intensity in linear scale ((b)–(f)) or in log scale ((g)–(i)). (Colors are visible in the online version of the article; http://dx.doi.org/10.3233/BSI-130057.)

**Fig. 3:**
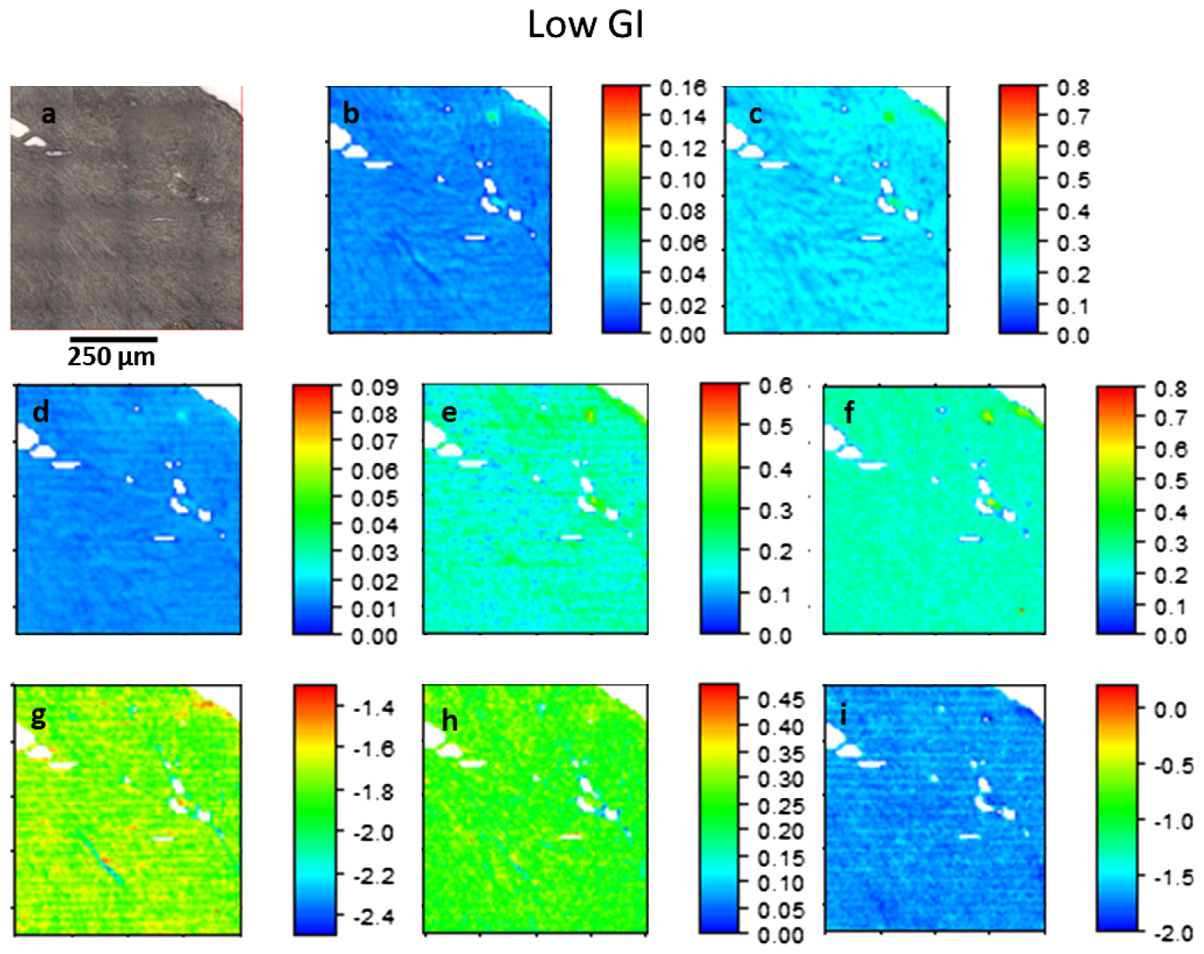
Bright field image and SR-FTIR heatmaps of a typical thin section of heart tissue from low GI-fed mice. ((a)–(i)) Same legend as Fig. 2. (Colors are visible in the online version of the article; http://dx.doi.org/10.3233/BSI-130057.)

Although the mean SR-FTIR absorbance intensities (Table 2) show that a high GI diet induces various degrees of compositional changes in mouse cardiac tissues there are marked regional heterogeneities, as indicated in the SR-FTIR spectroscopic maps of cardiac tissue from mice fed high (Fig. 2) or low GI (Fig. 3) diets. Here, each chemical image (Figs 2(b)–(f) and 3(b)–(f)) represents the integrated absorbance of a specific molecular band of the IR spectrum for each pixel. Figures 2(b) and 3(b), which were derived from the integrated absorbance of the *ν*(CH)_lipid_ bands (2800–3000 cm ^−1^) reveal the morphology and textures of myocardial tissue samples. A comparison of Figs 2(b) versus 3(b) shows that for mice fed high GI diets, there was almost a two-fold increase in the lipid absorbance in areas that correspond to capillaries and veins. Images derived from the sugar groups of carbohydrates (Figs 2(d)–3(d)), the *ν*(C–O) collagen bands (Figs 2(e)–3(e)), and the *ν*(C=O) lipid ester bands (Figs 2(f)–3(f)) show an elevated accumulation of carbohydrates, collagen and aggregates of lipid ester (presumably cholesteryl esters) in segments of the lipid-rich areas within perivenuous/venous tissues. The co-localization of the observed high intensities of the sugar groups of carbohydrates, C–O groups of collagen, and the C=O groups of lipid esters may reflect the enhanced deposition of glycated collagens and lipids [38,59], a marker of the presence of AGEs [24] and indicators of pathogenesis of arterial and myocardial stiffening of aging and diabetes [2]. The infrared spectra of glycation and AGE products exhibit strong absorption features from the *ν*(C–O; C–C)_sugar moieties_ vibrations and the *ν*(C–O–C)_sugar moieties + phospholipids_ in the 950–1180 cm^−1^ region [59,63]. The elevated ratio of the absorbance from the sugar moieties and phospholipids to the protein amide II shows the presence of AGEs. However, we cannot rule out the possibility that the co-located aggregates of lipid esters may also reflect the accumulation of the macrophage-derived foam cells [33,55].

For mice fed high GI diet, the lipid/protein ratio appeared to be uniformly higher relative to mice on a low GI diet (Figs 2(g) versus 3(g)). This was due to an increase in lipid content (Figs 2(b), 3(b)) and not a decrease in the protein content (Figs 2(c), 3(c)). Similarly, images of the CH_2_/CH_3_ ratio show significantly higher values (Figs 2(h) versus 3(h)). This increase in the CH_2_/CH_3_ ratio is consistent with the reported increase in the asymmetric and symmetric vibrations of CH_2_ and a decrease in the vibration of CH_3_ methylene groups of the fatty acids in cellular membranes of chronic hyperglycemic or diabetic heart tissues [69]. These increases in lipid/protein and the CH_2_/CH_3_ ratio suggest that a high GI diet disturbs lipid metabolism in ventricular myocardium, similar to those reported for DM [31,35,52,69].

### 3.3. AGEs hotspots

**Fig. 4:**
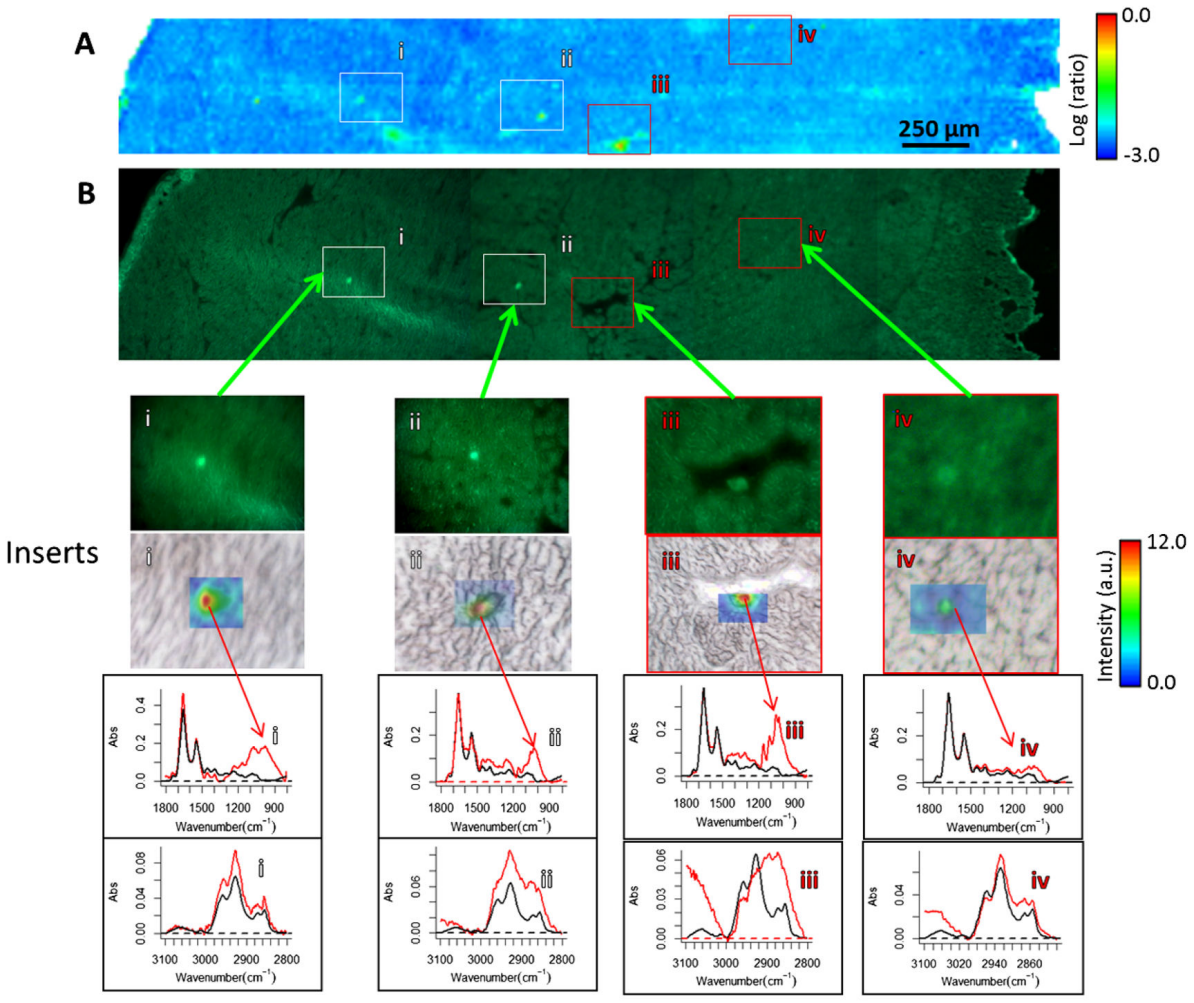
Comparative analysis of SR-FTIR and auto-fluorescence images shows different types of advanced glycation endproducts (AGEs) hotspots in cardiac tissue sections from high GI-fed mice. (A) The spatial distribution of the values of the ratio of carbohydrate band to protein ratio (in a logarithmic scale from −3 to 0.0), (B) the fluorescence images (excitation 450–490 nm, emission 500–550 nm) of the same region. White squares mark areas of fluorescence AGE hotspots (as elevated values of the ratio of carbohydrate to proteins and as bright spots in the fluorescence images), red squares mark areas of non-fluorescence AGE hotspots. Insets: heatmaps of Integrated absorbance of sugar moieties of carbohydrates (in linear scale from 0 to 12 a.u.). Below the heatmap insets are the corresponding SR-FTIR spectra of each “hotspot” in red (fingerprint region and lipid region) compared to the average spectrum of the HGI tissue (in black). (The colors are visible in the online version of the article; http://dx.doi.org/10.3233/BSI-130057.)

A spectroscopic mapping of the carbohydrate/protein ratio over a larger area of the same cardiac tissue (Fig. 4(a)) revealed glycation hot spots in some veins and capillaries (Fig. 4(a) versus insert 1). Within these microscopic hotspots the values of the carbohydrate/protein ratio often reached between 0.7 and 1.0, reflecting the high accumulations of protein–carbohydrate conjugates. These glycation hot spots, as marked in Fig. 4(a), typically ranged from several to tens microns in size (inserts 4(i)–(iv)). The fluorescence image (excitation at 330–385 nm/emission at *>*420 nm) of the same sample section demonstrates the presence of the fluorescent AGE-related pentosidine or pentosidine-like cross-links between the arginine and lysine residues (Fig. 4(b), inserts (i) and (ii)), and the nonfluorescence N*-(carboxymethyl)lysine-like AGE (Fig. 4(b), inserts 4(iii) and (iv)) [20,21,23,27–29,65,66].

Comparison of the SR-FTIR spectra of all AGE hotspots with the mean SR-FTIR spectra shows little significant changes in the position of protein amide I band (*~*1648 cm^−1^), a measure of the protein secondary structure as defined by patterns of hydrogen bonds between the peptides. This implies a surprising conservation of the protein structure in spite of glycation. Meanwhile, the increase in absorption intensity in the amide I and amide II bands may reflect discrete conformational changes in tertiary structure of proteins at the AGE hotspots. The most changes were observed in the 1200–900 cm^−1^ region (for example, insets in Fig. 4). Here the absorbance bands arise from composite modes of vibrations of the sugar ring overlapping with stretching vibrations of the side group (C–OH) and with the (C–O–C) glycosidic bond vibration of the sugar moieties of glycated proteins [38,59] including proteoglycan [56]. A deeper analysis reveals almost all AGE hotspots’ absorption bands in this 1200–900 cm^−1^ region were dominated by the glucose configuration (Fig. 4, inserts (i)–(iv)): *~*1160 cm^−1^ from the (C–O–C) glycosidic bond, *~*1105 cm^−1^ and *~*1079 cm^−1^ from the (COH) group vibrations on the equatorial and the axial position, and the lowest frequency maximum intensity at *~*1030 cm^−1^. Along with spectral features indicating protein-sugar aggregates it is possible to identify infrared signals that can be related to oxidative stress processes. An increase in the intensity of ester carbonyl groups (R–O–C=O) at *~*1735 cm^−1^ [41,50], often co-localized with fluorescence hotspots, could be due to accumulation of lipid peroxidation products such as malondialdehyde, lipid aldehyde, and alkyl radicals, which are known to be present in pathologies such as diabetes [4]. The observed increase in the olefinic band (=CH) at *~*3012 cm^−1^ in Fig. 4 (insert (i)) implies the presence of double bonds is associated with the release of lipid peroxidation products such as malondialdehyde, lipid aldehyde, and alkyl radicals into the extra- or intracellular site of the cell [64]. These released products could cause apoptosis or necrotic cell death [26].

### 3.4. Histopathological assessment of cardiac tissues

**Fig. 5:**
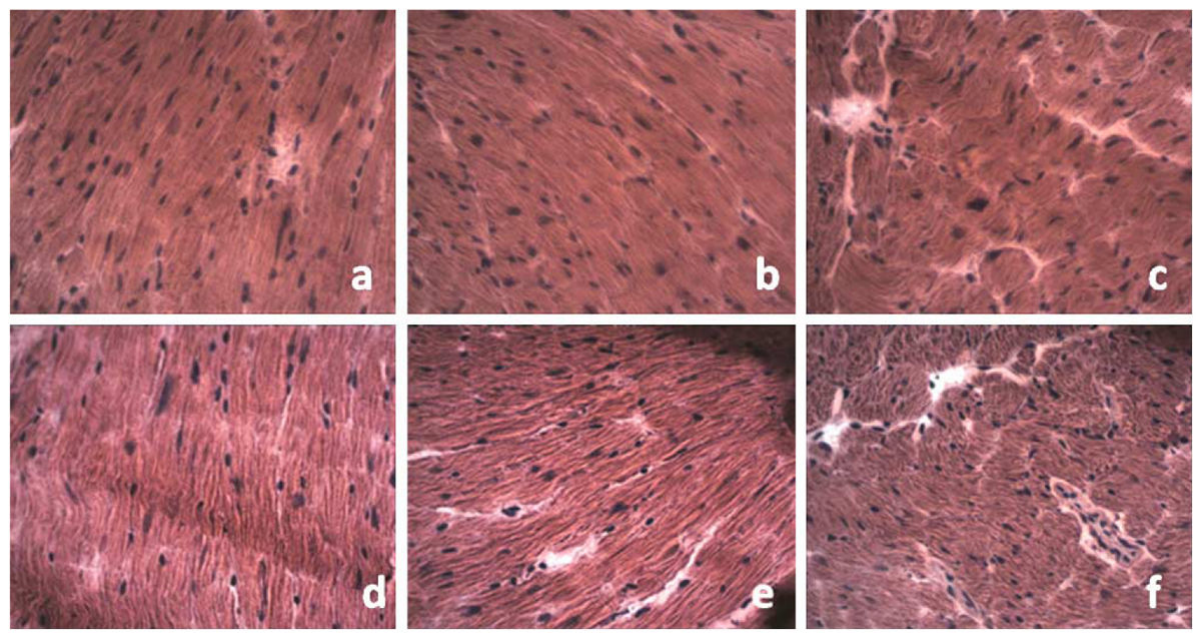
Histological assessment of differently oriented cardiac tissue sections of mice fed high ((a)–(c)) or low ((d)–(f)) GI diets. Representative images of H&E staining (all magnifications: 40*×*, each image is 225 *×* 180 μm^2^). Typical views (a:d = longitudinal, b:e = oblique, c:f = transverse cross sections) of cardiac tissues from C57BL/6 mice fed high GI (*top*) and low GI (*bottom*) diets, showing layered sheets of cardiac muscle cells (spindle-shaped and pink with blued stained multiple nucleoli within the center of each myocyte), separated by “interstitial tissue voids” (clear) between cardiac muscle cell fibers. Nuclear material was stained with hematoxylin (deep blue-purple color) and cytoplasmic material including connective tissue and collagen was stained with eosin (a bright pink color). (The colors are visible in the online version of the article; http://dx.doi.org/10.3233/BSI-130057.)

Histological characteristics of the even-numbered cardiac tissue sections were evaluated for mice fed high (Fig. 5(A)) and low (Fig. 5(B)) GI diets. Hematoxylin, a basic dye, stains the nuclear material to yield a deep blue-purple color. The heart is rich in blood vessels as well as cardiac muscle and collagen. Eosin, an acidic dye, counterstains cytoplasmic materials including connective tissue and collagen to yield a bright pink color. The eosin demonstrates the normally well-defined cross-striations of the car-diomyocyte I- and H bands which correspond to overlapping regions of actin thin filaments and thick myosin fibers within each sarcomere. Various hues were present in the sample, including yellow and brown due to intrinsic pigments. Hydrophobic structures such as Golgi membranes or lipids remain clear. H&E stained sections from mice fed with high GI diets typically showed cell enlargement or cardiac hypertrophy with the loss of definition of cardiomyocyte cross-striation pattern. This increase in cell size in the absence of cell division (cardiac hypertrophy) and the loss of normal actin and myosin microstructure could be an indicator of ventricular dysfunction due to a high GI diet. It is known that with chronically high levels of glucose there is significant increased risk of congestive heart failure [1,53]. Cardiomyocyte hypertrophy coupled to disorganization of actin and myosin filaments we have observed in the current model appears to be consistent with previous clinical studies which demonstrated that hearts suffering from glycemia-related cardiac impairments have altered wall thickness and abnormal ventricular volumes [16,47].

## 4. Discussion

The growing obesity, and diabetes epidemics make it imperative to develop new means to diagnose and treat these and associated diseases including macular degeneration and CVD. Considerable literature indicates the dangers of diets that are high in rapidly digested starches, with respect to risk for these diseases. These are also called high glycemic index (GI) diets. These include white breads, sodas, and sweet deserts. Lower glycemia diets are usually rich in fruits, non-starchy vegetables, and whole grain products.

In this study, we compared and contrasted through a well-characterized amylopectin-based dietary mouse model, the effects of a high GI diet to those of a low GI diet on the biochemical composition and microstructure of the heart. The improved spatial resolution and signal-to-noise for SR-FTIR spectromicroscopy enabled us to obtain a molecular-level observation of diet-related changes within unfixed fresh frozen histologic sections of mouse cardiac tissue. Serial cryosections of cardiac tissue for the combined SR-FTIR and fluorescence imaging alternated with adjacent hematoxylin and eosin (H&E) stained sections allowed not only fine-scale chemical analyses of glycogen and glycolipid accumulation along a vein as well as protein and lipid glycation hotspots co-localizing with elevated collagen but also the tracking of morphological differences occurring in tandem with these chemical changes. As a result of the bright synchrotron infrared photon source coupling, we were able to provide significant molecular evidence for elucidating and supporting a positive correlation between protein and lipid glycation (AGEs) and collagen accumulation in our mouse model.

We have provided direct molecular evidence that support the notion that consumption of high GI diets can increase the risk cardiovascular disease [49,51] through the observed presence of both the fluorescent pentosidine or pentosidine-like cross-linkings between the arginine and lysine residues in collagen, and the nonfluorescence N•-(carboxymethyl)lysine-like AGEs. Similar to chronic hyperglycemia or high blood sugar from diabetes mellitus, our SR-FTIR and fluorescence imaging analyses show that chronic high GI diet enhances the production of AGEs and increases the risk for cardiovascular disease in our mouse model. Our results support the epidemiologic data indicating that people who consume low glycemic index (GI) diets have lower blood sugar [58] and are at reduced risk for the onset and progression of age-related cardiovascular disease [42].

## Figures and Tables

**Table 1:** Main infrared absorbance band integrals of proteins, lipids and carbohydrates

#	Primary contributing biomolecules	Wavenumber (cm^−1^)	Band assignments
1	Unsaturated lipids	*~*3010	*ν*(=C–H)
2	Saturated lipids	*~*2965	*ν*_as_(C–H) of methyl group (CH_3_)
		*~*2925	*ν*_as_(C–H) of methylene group (CH_2_)
		*~*2875	*ν*_s_(C–H) of methyl group (CH_3_)
		*~*2845	*ν*_s_(C–H) of methylene group (CH_2_)
3	Lipid esters	*~*1735	*ν* (C=O) of esters
4	Amino acid side chains, nucleic acids	*~*1715–1690	*ν* (C=O) of carbonic acids
5	Proteins	*~*1695–1610	amide I (predominantly *ν* (C=O) coupled with *ν* (C–N), *δ*(N–H))
		*~*1550	amide II (*ν* (C–N) coupled with *δ*(N–H))
		*~*1310–1240	amide III
6	Nucleic acids, phosphorylated proteins, phospholipids	*~*1250–1220	*ν*_as_(P=O) of phosphodiesters (PO_2_^−^)
7	Collagen	*~*1210–1200	*ν* (C–O) band centered at *~*1204 cm^−1^
8	Carbohydrates, nucleic acids, phospholipids and proteins	*~*1130–900	*ν* (C–O–C), *ν* (C–O), *ν* (C–C), *ν* (C–O–P), *ν* (P–O–P)
9	Nucleic acids, phosphorylated proteins, phospholipids	*~*1080	*ν*_s_(P=O) of phosphodiesters (PO_2_^−^)

*Notes*: Adapted from [19]. *ν*– stretching, s – symmetric, as – asymmetric.

**Table 2:** Infrared absorbance band integrals of macromolecules proteins, lipids and carbohydrates within the heart tissue of mice fed with a high GI versus a low GI diet

	High GI diet (mean *±* s.d.)*	Low GI diet (mean *±* s.d.)*	%
Proteins	24.350 *±* 3.108	21.022 *±* 2.867	+7
Lipids	3.030 *±* 0.534	2.232 *±* 0.411	+15
Carbohydrates	1.724 *±* 0.354	1.392 *±* 0.179	+11
Collagen	0.201 *±* 0.046	0.185 *±* 0.037	+4
Lipid esters	0.314 *±* 0.056	0.252 *±* 0.035	+11

* Both the mean and standard deviation values are expressed in absorbance units (a.u.). The mean values are the average of the integral absorbance value for 7839 spectra from high GI diet mice and 7092 spectra from low GI diet mice. The high GI/-low GI diet Tukey test *p*-values are 3 × 10^−8^.

## References

[1] Anversa P, Nadal-Ginard B (2002). Myocyte renewal and ventricular remodelling. Nature.

[2] Aronson D (2003). Cross-linking of glycated collagen in the pathogenesis of arterial and myocardial stiffening of aging and diabetes. J Hypertens.

[3] Aronson D, Rayfield EJ (2002). How hyperglycemia promotes atherosclerosis: molecular mechanisms. Cardiovasc Diabetol.

[4] Baynes JW, Thorpe SR (1999). Role of oxidative stress in diabetic complications: a new perspective on an old paradigm. Diabetes.

[5] Beekes M, Lasch P, Naumann D (2007). Analytical applications of Fourier transform-infrared (FT-IR) spectroscopy in microbiology and prion research. Veterinary Microbiology.

[6] Bonda M (2011). Synchrotron infrared microspectroscopy detecting the evolution of Huntington’s disease neuropathology and suggesting unique correlates of dysfunction in white versus gray brain matter. Analytical Chemistry.

[7] Boskey A, Camacho NP (2007). FT-IR imaging of native and tissue-engineered bone and cartilage. Biomaterials.

[8] Bunn HF (1981). Nonenzymatic glycosylation of protein: relevance to diabetes. Am J Med.

[9] Camacho NP, Landis WJ, Boskey AL (1996). Mineral changes in a mouse model of osteogenesis imperfecta detected by Fourier transform infrared microscopy. Connective Tissue Research.

[10] Chen L (2012). Synchrotron infrared measurements of protein phosphorylation in living single PC12 cells during neuronal differentiation. Analytical Chemistry.

[11] Chiarelli F (1999). Advanced glycation end products in children and adolescents with diabetes: relation to glycemic control and early microvascular complications. J Pediatr.

[12] Chiu CJ (2009). Does eating particular diets alter the risk of age-related macular degeneration in users of the age-related eye disease study supplements?. Br J Ophthalmol.

[13] Chiu CJ (2009). Dietary compound score and risk of age-related macular degeneration in the age-related eye disease study. Ophthalmology.

[14] Colarusso P (1998). Infrared spectroscopic imaging: From planetary to cellular systems. Applied Spectroscopy.

[15] Didonna A (2011). Infrared microspectroscopy: a multiple-screening platform for investigating single-cell biochemical perturbations upon prion infection. ACS Chem Neurosci.

[16] Doi K (2001). Alteration of antioxidants during the progression of heart disease in streptozotocin-induced diabetic rats. Free Radic Res.

[17] Dumas P (2006). Synchrotron infrared microscopy at the French Synchrotron Facility SOLEIL. Infrared Physics & Technology.

[18] Fernandez DC (2005). Infrared spectroscopic imaging for histopathologic recognition. Nature Biotechnology.

[19] Freeman NK (1964). Simultaneous determination of triglycerides and cholesterol esters in serum by infrared spectrophotometry. J Lipid Res.

[20] Fu MX (1994). Glycation, glycoxidation, and cross-linking of collagen by glucose. Kinetics, mechanisms, and inhibition of late stages of the Maillard reaction. Diabetes.

[21] Fu MX (1996). The advanced glycation end product, Nepsilon-(carboxymethyl)lysine, is a product of both lipid peroxidation and glycoxidation reactions. Journal of Biological Chemistry.

[22] Fuchs RK (2008). *In situ* examination of the time-course for secondary mineralization of haversian bone using synchrotron Fourier transform infrared microspectroscopy. Matrix Biol.

[23] Goh SY, Cooper ME (2008). Clinical review: The role of advanced glycation end products in progression and complications of diabetes. J Clin Endocrinol Metab.

[24] Goldin A (2006). Advanced glycation end products: sparking the development of diabetic vascular injury. Circulation.

[25] Hackett MJ (2013). Subcellular biochemical investigation of Purkinje neurons using synchrotron radiation Fourier transform infrared spectroscopic imaging with a focal plane array detector. ACS Chem Neurosci.

[26] Hampton MB, Orrenius S (1997). Dual regulation of caspase activity by hydrogen peroxide: implications for apoptosis. FEBS Lett.

[27] Hartog JW (2007). Advanced glycation end-products (AGEs) and heart failure: pathophysiology and clinical implications. Eur J Heart Fail.

[28] Hartog JW (2008). Skin-autofluorescence, a measure of tissue advanced glycation end-products (AGEs), is related to diastolic function in dialysis patients. J Card Fail.

[29] Hegab Z (2012). Role of advanced glycation end products in cardiovascular disease. World J Cardiol.

[30] Heraud P (2010). Early detection of the chemical changes occurring during the induction and prevention of autoimmune-mediated demyelination detected by FT-IR imaging. Neuroimage.

[31] Heyliger CE, Rodrigues B, McNeill JH (1986). Effect of choline and methionine treatment on cardiac dysfunction of diabetic rats. Diabetes.

[32] Holman HY (2000). IR spectroscopic characteristics of cell cycle and cell death probed by synchrotron radiation based Fourier transform IR spectromicroscopy. Biopolymers.

[33] Holman HY (2008). Mid-infrared reflectivity of experimental atheromas. J Biomed Opt.

[34] Holman HY (2010). Synchrotron IR spectromicroscopy: Chemistry of living cells. Analytical Chemistry.

[35] Hsiao YC (1987). Ultrastructural alterations in cardiac muscle of diabetic BB Wistar rats. Virchows Arch A Pathol Anat Histopathol.

[36] Jamin N (1998). Highly resolved chemical imaging of living cells by using synchrotron infrared microspectrometry. Proc Natl Acad Sci USA.

[37] Jenkins DJ (1981). Glycemic index of foods: a physiological basis for carbohydrate exchange. Am J Clin Nutr.

[38] Khajehpour M, Dashnau JL, Vanderkooi JM (2006). Infrared spectroscopy used to evaluate glycosylation of proteins. Anal Biochem.

[39] Kidder LH (1997). The exploration of lipid domain formation using classical and imaging vibrational spectroscopic methods. Biophysical Journal.

[40] Kretlow A (2008). Changes in protein structure and distribution observed at pre-clinical stages of scrapie pathogenesis. Biochimica et Biophysica Acta – Molecular Basis of Disease.

[41] Lasch P (2001). Hydrogen peroxide-induced structural alterations of RNAse A. J Biol Chem.

[42] Leeds AR (2002). Glycemic index and heart disease. Am J Clin Nutr.

[43] Leskovjan AC, Kretlow A, Miller LM (2010). Fourier transform infrared imaging showing reduced unsaturated lipid content in the hippocampus of a mouse model of Alzheimer’s disease. Analytical Chemistry.

[44] Lester DS (1998). Infrared microspectroscopic imaging of the cerebellum of normal and cytarabine treated rats. Cellular and Molecular Biology.

[45] Levin IW (1998). Biomembrane and cellular domains: Infrared spectroscopic and imaging studies. Biophysical Journal.

[46] Lewis EN (1997). Infrared spectroscopic imaging studies of brain tissue derived from the mutant Niemann Pick C mouse. Biophysical Journal.

[47] Litwin SE (1990). Abnormal cardiac function in the streptozotocin-diabetic rat. Changes in active and passive properties of the left ventricle. J Clin Invest.

[48] Liu KZ, Dixon IM, Mantsch HH (1999). Distribution of collagen deposition in cardiomyopathic hamster hearts determined by infrared microscopy. Cardiovasc Pathol.

[49] Liu S (1999). Whole-grain consumption and risk of coronary heart disease: results from the Nurses’ Health Study. Am J Clin Nutr.

[50] Mamarelis I (2010). Oxidative stress and atherogenesis. An FT-IR spectroscopic study. In Vivo.

[51] McKeown NM (2009). Dietary carbohydrates and cardiovascular disease risk factors in the Framingham offspring cohort. J Am Coll Nutr.

[52] McNeill JH (1996). Role of elevated lipids in diabetic cardiomyopathy. Diabetes Res Clin Pract.

[53] Nakamura K (1998). Inhibitory effects of antioxidants on neonatal rat cardiac myocyte hypertrophy induced by tumor necrosis factor-alpha and angiotensin II. Circulation.

[54] Nathan DM (2005). Intensive diabetes treatment and cardiovascular disease in patients with type 1 diabetes. N Engl J Med.

[55] Palombo F (2009). Application of Fourier transform infrared spectroscopic imaging to the study of effects of age and dietary L-arginine on aortic lesion composition in cholesterol-fed rabbits. Journal of the Royal Society Interface.

[56] Potter K (2001). Imaging of collagen and proteoglycan in cartilage sections using Fourier transform infrared spectral imaging. Arthritis Rheum.

[57] Romeo MJ, Diem M (2005). Infrared spectral imaging of lymph nodes: Strategies for analysis and artifact reduction. Vibrational Spectroscopy.

[58] Rovner AJ, Nansel TR, Gellar L (2009). The effect of a low-glycemic diet vs a standard diet on blood glucose levels and macronutrient intake in children with type 1 diabetes. J Am Diet Assoc.

[59] Roy R, Boskey A, Bonassar LJ (2010). Processing of type I collagen gels using nonenzymatic glycation. J Biomed Mater Res A.

[60] Ruberg FL (2007). Myocardial lipid accumulation in the diabetic heart. Circulation.

[61] Ruppel ME, Burr DB, Miller LM (2006). Chemical makeup of microdamaged bone differs from undamaged bone. Bone.

[62] Sato T (2009). Effects of high-AGE beverage on RAGE and VEGF expressions in the liver and kidneys. European Journal of Nutrition.

[63] Scott DA (2010). Diabetes-related molecular signatures in infrared spectra of human saliva. Diabetology & Metabolic Syndrome.

[64] Severcan F (2005). Rapid monitoring of diabetes-induced lipid peroxidation by Fourier transform infrared spectroscopy: evidence from rat liver microsomal membranes. Anal Biochem.

[65] Shapiro BP (2008). Advanced glycation end products accumulate in vascular smooth muscle and modify vascular but not ventricular properties in elderly hypertensive canines. Circulation.

[66] Shaw JN, Baynes JW, Thorpe SR (2002). N epsilon-(carboxymethyl)lysine (CML) as a biomarker of oxidative stress in long-lived tissue proteins. Methods Mol Biol.

[67] Stultz CM, Edelman ER (2003). A structural model that explains the effects of hyperglycemia on collagenolysis. Biophysical Journal.

[68] Taylor A (1995). Dietary calorie restriction in the Emory mouse: effects on lifespan, eye lens cataract prevalence and progression, levels of ascorbate, glutathione, glucose, and glycohemoglobin, tail collagen breaktime, DNA and RNA oxidation, skin integrity, fecundity, and cancer. Mech Ageing Dev.

[69] Toyran N (2006). Early alterations in myocardia and vessels of the diabetic rat heart: an FTIR microspectroscopic study. Biochem J.

[70] Turker S (2012). Application of infrared spectroscopy in the study of neurological diseases. Biomedical Spectroscopy and Imaging.

[71] Uchiki T (2012). Glycation-altered proteolysis as a pathobiologic mechanism that links dietary glycemic index, aging, and age-related disease (in nondiabetics). Aging Cell.

[72] Weikel KA (2012). Natural history of age-related retinal lesions that precede AMD in mice fed high or low glycemic index diets. Invest Ophthalmol Vis Sci.

[73] Wood BR (2004). Methanol formation on Fe/Al-MFI via the oxidation of methane by nitrous oxide. Journal of Catalysis.

